# Role of long noncoding RNA UCA1 as a common molecular marker for lymph node metastasis and prognosis in various cancers: a meta-analysis

**DOI:** 10.18632/oncotarget.12463

**Published:** 2016-10-04

**Authors:** Anbang He, Rong Hu, Zhicong Chen, Xinhui Liao, Jianfa Li, Dailian Wang, Zhaojie Lv, Yuchen Liu, Feng Wang, Hongbing Mei

**Affiliations:** ^1^ Department of Urology, Shenzhen Second People's Hospital, The First Affiliated Hospital of Shenzhen University, Shenzhen, Guangdong, China; ^2^ Key Laboratory of Medical Reprogramming Technology, Shenzhen Second People's Hospital, The First Affiliated Hospital of Shenzhen University, Shenzhen, China; ^3^ Shantou University Medical College, Shantou, Guangdong, China; ^4^ Guangzhou Medical University, Guangzhou, Guangdong, China

**Keywords:** UCA1, lncRNA, prognosis, survival, lymph node metastasis

## Abstract

Accumulating evidences indicated that UCA1 expression was up-regulated in various cancers, and high UCA1 expression was correlated with metastasis and prognosis. This meta-analysis collected all eligible studies and explored the relationships between UCA1 expression and lymph node metastasis (LNM) or overall survival (OS). Literature collection was performed by using electronic databases PubMed, Cochrane Library, and Web of Science (up to June 13, 2016). According to the inclusion and exclusion criteria, twelve studies were included in the meta-analysis. The result showed that high UCA1 expression was correlated with more LNM (OR=2.50, 95 %CI: 1.58-3.96, p<0.0001) in a random-effects model (I2=45 %, p=0.08) and could predict poor OS in cancer patients, with pooled hazard ratio (HR) of 1.65 [95% confidence interval (CI) 1.44-1.88, p<0.00001] indicated by a fixed-effects model (I2=35%, p=0.11). In conclusion, the present meta-analysis demonstrated that high expression of UCA1 might serve as a common molecular marker for predicting lymph node metastasis and prognosis in various cancers.

## INTRODUCTION

With increasing incidence and mortality in each year, cancer is becoming the leading cause of death worldwide and is a major public health problem. In the United States, there will be 1,685,210 new cancer cases and 595,690 cancer deaths in 2016 [1]. For patients in early stage of cancers without lymph node metastasis or distant metastasis, some treatments are available, such as surgery, chemotherapy and radiation therapy. However, in patients with lymph node metastasis or distant metastasis, there are no available therapies. Therefore, finding a common molecular cancer marker for predicting lymph node metastasis and prognosis is indispensable for observing the progression of cancers.

With the rapid development of second-generation sequencing technology, lots of long noncoding RNAs (lncRNAs) have been found to be dysregulated in expression and involved in the development of various human diseases, particularly in cancers [2]. Accumulating evidences reveal that lncRNAs play vital regulatory roles in diverse cellular processes, such as regulation of gene expression, posttranslational processing and tumorigenesis [3]. Urothelial Carcinoma Associated 1 (UCA1), a 2314-bp lncRNA encoded on human chromosome 19, was firstly identified in bladder cancer [4]. A large amount of studies have demonstrated that UCA1 expression was up-regulated in many cancers, such as hepatocellular cancer, colorectal cancer, gastric cancer, esophageal squamous cell carcinoma and lung cancer, and that high UCA1 expression was strongly correlated with clinicopathologic characteristics, including lymph node metastasis and overall survival [5–10]. According to these findings, we carried out this meta-analysis to explore the relationships between UCA1 expression and lymph node metastasis or overall survival, and to evaluate whether UCA1 could serve as a common molecular marker for LNM and OS.

## RESULTS

### Characteristics of eligible studies

**Figure 1:**
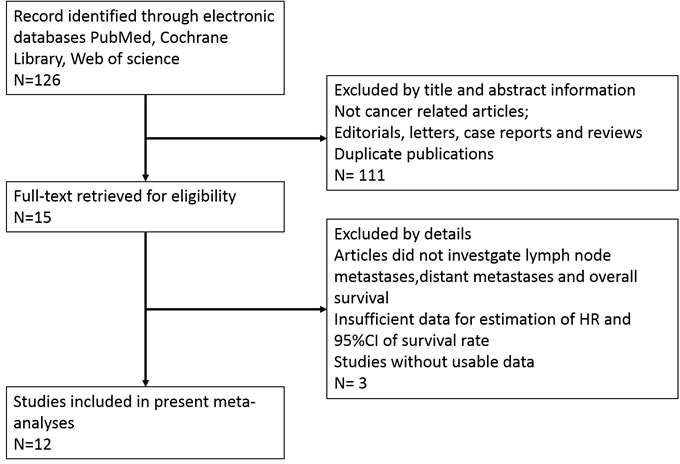
The flow diagram of this meta-analysis

As shown in the flow diagram (Figure 1), a total of 126 published articles were revealed in our initial search by using the keywords. After screening the title and abstract carefully, 111 articles were excluded. After further inspection of the full articles, 2 were excluded because there is no available information in their records. In the 13 potential candidate studies, 1 study was excluded because of inadequate data of HR with 95 % CI. Finally, according to the criteria for selection, a total of 12 studies were eligible [5–16].

These studies included a total of 986 patients. The mean patient sample size was 82.17 (range from 40 to 117). Among the twelve studies, one focused on esophageal squamous cell carcinoma, one on non-small cell lung cancer, one on lung cancer, one on colon cancer, one on gastric cancer, one on hepatocellular carcinoma, one on prostate cancer, two on ovarian cancer and three on colorectal cancer. All the diagnoses of lymph node metastasis were based on pathology. In all of the studies, the patients were divided into two groups: high and low expression of UCA1. All studies used qRT-PCR to detect UCA1 expression. The main characteristics of the eligible studies were summarized in Table 1 and Table 2.

**Table 1:** Characteristics of studies in this meta-analysis

Author	Year	Country	Tumor type	Sample size(n)	UCA1 expression				Method
					High expression	High with LNM	Low expression	Low with LNM	
Li	2014	China	ESCC	90	41	22	49	12	RT-qPCR
Nie	2016	China	NSCLC	112	39	14	73	21	RT-qPCR
Wang	2015	China	LC	60	36	26	24	8	RT-qPCR
Zhang	2016	China	OC	110	57	26	53	12	RT-qPCR
Yang	2016	China	OC	53	27	13	26	5	RT-qPCR
Ni	2015	China	CRC	54	27	12	27	5	RT-qPCR
Tao	2015	China	CC	80	20	13	60	21	RT-qPCR
Zheng	2015	China	GC	112	56	35	56	37	RT-qPCR

ESCC esophageal squamous cell carcinoma, NSCLC non-small cell lung cancer, LC lung cancer, OC Ovarian cancer, CRC colorectal cancer, CC colon cancer, GC gastric cancer, qRT-PCR quantitative real-time PCR, LNM lymph node metastasis.

**Table 2:** Overall survival of the eligible studies in this meta-analysis

Study	Year	Disease	Number	MALAT1 assay	Survival analysis	Multivariate analysis	HR statistic	Hazard ratios(95% CI)	Follow-up, moths
Zheng	2015	GC	112	RT-qPCR	OS	Yes	Data in paper	2.351(1.222-4.521)	60
Zhang	2016	OC	117	RT-qPCR	OS	Yes	Data in paper	1.688(1.005-2.834)	>60
Yang	2016	OC	53	RT-qPCR	OS	Yes	Data in paper	6.318(1.119-35.679)	NA
Bian	2016	CRC	90	RT-qPCR	OS	Yes	Data in paper	2.395 (1.044-5.495)	>75
WangF	2015	HCC	98	RT-qPCR	OS	Yes	Data in paper	1.859 (1.077-3.210)	60
Nie	2016	NSCLC	112	RT-qPCR	OS	Yes	Data in paper	1.409(1.077-1.844)	>60
Tao	2015	CC	80	RT-qPCR	OS	Yes	Data in paper	2.002 (1.007-3.981)	60
Ni	2015	CRC	54	RT-qPCR	OS	NO	Survival curve	3.137(1.1707-8.4056)	NA
Na	2015	PC	40	RT-qPCR	OS	Yes	Survival curve	1.52(1.2309- 1.8769)	60
Han	2014	CRC	80	RT-qPCR	OS	NO	Survival curve	0.190 (0.0372-0.9709)	NA
WangH	2015	LC	60	RT-qPCR	OS	Yes	Data in paper	1.936 (1.062-3.258)	60
Li	2014	ESCC	90	RT-qPCR	OS	Yes	Data in paper	2.627(1.416-5.874)	60

ESCC esophageal squamous cell carcinoma, NSCLC non-small cell lung cancer, LC lung cancer, OC ovarian cancer, CRC colorectal cancer, CC colon cancer, GC gastric cancer, HCC hepatocellular carcinoma, PC prostate cancer, qRT-PCR quantitative real-time PCR,NA not available.

### Meta-analysis results

#### Association between UCA1 and LNM

**Figure 2:**
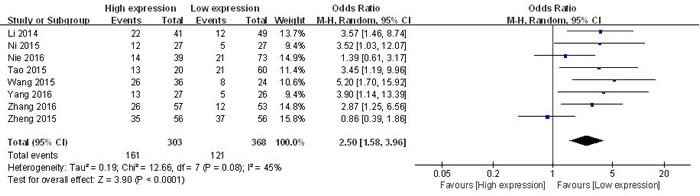
Forest plot for the association between UCA1 expression levels with LNM

All of the eligible studies reported the LNM of 671 patients based on different UCA1 expression levels. The random-effects model was adopted because of the significant between-study heterogeneity (I^2^ = 45 %, *p* = 0.08). The odds ratios, expressed as high UCA1 expression group *versus* low UCA1 expression group, was 2.50 (CI 95 % 1.58-3.96, *p* < 0.0001 in random-effects model) (Figure 2). The result revealed that patients with high UCA1 expression in tumor tissues were more prone to LNM.

#### Association between UCA1 and OS

**Figure 3:**
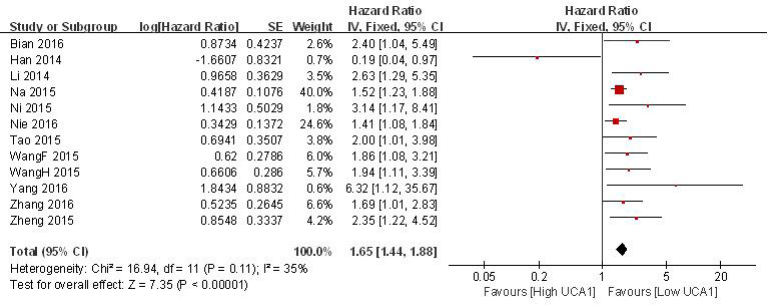
Forest plot of the pooled HRs of elevated UCA1 expression for OS for the included studies

Data of pooled HRs and 95 %CI of overall survival were collected from the 12 eligible studies. The fixed-effects model was used as the small heterogeneity (I_2_ = 35 %, *p* = 0.11). Analysis showed a pooled OR of 1.65 with 95 %CI 1.44-1.88 (*p* < 0.00001) (Figure 3). Compared with low UCA1 expression group, high UCA1 expression group showed a statistically significant reduction in OS and a correlation with a worse survival.

#### Sensitivity analysis

**Figure 4:**
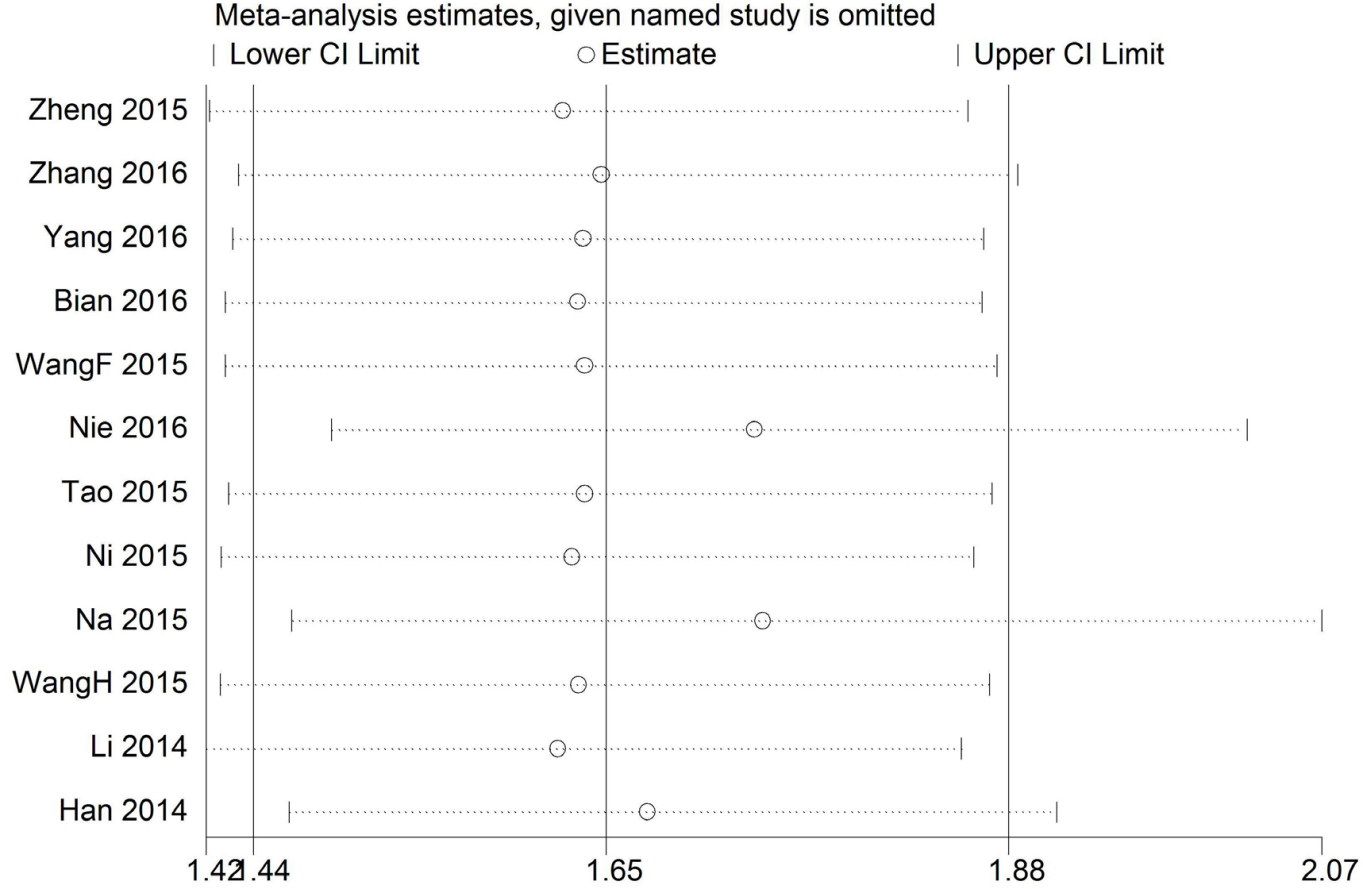
Result of sensitivity analysis in OS group

Sensitivity analysis was performed to assess the effect of a single study on the overall meta-analysis results by omitting one study at a time in total population. When each study was excluded sequentially, none of the results were significantly altered each time (Figure 4). Because of the small number of studies, the sensitivity analysis was not analyzed in LNM group.

#### Publication bias

The publication bias of the present meta-analysis was evaluated by Egger's test. In the OS group, the Egger's test did not display obvious publication bias for the HR evaluations of OS (Egger's test, *t* = 1.42, *p* = 0.187) (Figure 4). As for LNM group, the publication bias was not analyzed because of the small number of studies.

## DISCUSSION

UCA1 has been recently found to be up-regulated in several cancers, contributing to tumor proliferation, apoptosis, metastasis and survival [4]. UCA1 increases cell proliferation through KLF4-KRT6/13 signaling pathway and FGFR1/ERK signaling pathway, respectively, in prostate cancer [11] and hepatocellular carcinoma [5]. Upregulated UCA1 is associated with tumor progression through targeting miR-193a-3p, miR-204-5p in non-small cell lung cancer [14] and colorectal cancer [7] individually. Moreover, UCA1 functions as a competing endogenous RNA to suppress epithelial ovarian cancer metastasis [15].UCA1, significantly associated with LNM and OS, may function as on oncogene and a potential indicator of prognosis in some cancers, including colorectal cancer, esophageal squamous cell carcinoma, prostate cancer, hepatocellular carcinoma and ovarian cancer [5–16].

This meta-analysis explored the relationships between UCA1 expression levels and LNM, DM and OS. The results indicated that high UCA1 expression was significant correlated with LNM (OR = 2.50, 95 %CI: 1.58-3.96, *p* < 0.0001), and poor OS (HR = 1.65, 95 %CI: 1.44-1.88, *p* < 0.00001). Through the above analysis, we found that high expression of UCA1 might serve as a common molecular marker for lymph node metastasis and poor prognosis in various cancers. Nevertheless, several limitations in the present meta-analysis should be emphasized. First, only twelve studies were included in this meta-analysis; it has weakened the reliability of this meta-analysis' results. So larger-size and better design studies are needed to be conducted to confirm our results. Second, because of different types of cancers, the cutoff value of UCA1 expression was different in each eligible study. Third, patients included in this present meta-analysis were all Asians. Because of this, our results may just account for patients from Asia. Fourth, many included studies reported positive results because negative results would have little chance to be published.

In conclusion, the meta-analysis offers evidence that up-regulated UCA1 is significantly corrected with LNM and poor OS in patients with various cancers. UCA1 may serve not only as a molecular marker for LNM, but also as a prognostic factor for patients with various cancers. Nevertheless, larger-size and better design studies are to be conducted to confirm its precise role among other well-known molecular markers and prognostic factors.

**Figure 5:**
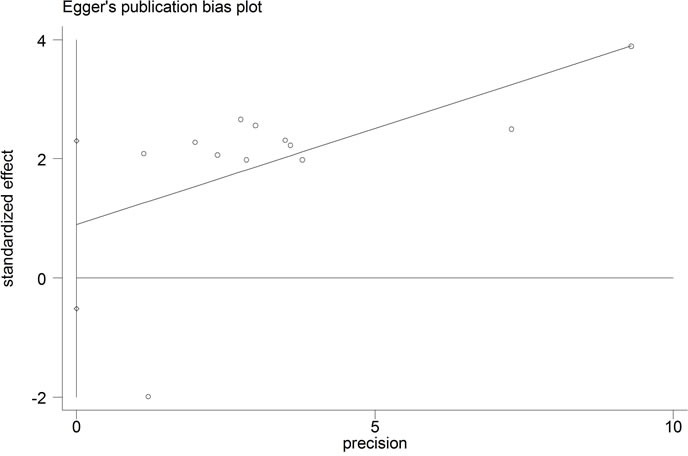
Funnel plot analysis of potential publication bias in OS group (Egger's test)

## MATERIALS AND METHODS

### Literature collection

We exhaustively searched electronic databases PubMed, Cochrane Library, and Web of Science, by using “UCA1 or urothelial carcinoma associated 1” as the keyword, in order to identify potentially relevant studies. The last update of searching time was June 13, 2016.

### Inclusion and exclusion criteria

Inclusion criteria are as the following: (1) evaluation of a link between UCA1 expression and prognosis of patients with any type of cancer. (2) patients were grouped according to the expression levels of UCA1.(3) related clinicopathologic parameters were described, such as lymph node metastasis.(4) reporting of outcomes, including overall survival (OS). (5) studies containing sufficient data for the computation of odds ratios (OR) and corresponding 95 % confidence intervals (CI). Exclusion criteria are as the following: (1) nonhuman research, letters, editorials, expert opinions, case reports and reviews. (2) studies without usable data. (3) duplicate publications.

### Quality assessment

The Newcastle-Ottawa Scale (NOS) was applied to assess the quality of all included studies by three investigators (ABH, ZCC and DLW) independently. All eligible studies were assessed to be of high quality by using the Newcastle-Ottawa Scale (NOS).

### Date extraction

Data extraction from the eligible studies were carried out independently by three authors (HAB, CZC and LXH), according to the inclusion and exclusion criteria. Disagreements were discussed with two investiga­tors (WDL,LJF) in conference. For each eligible study, the following information was collected: first author, publication date, country of origin, tumor type, total number of patients, number of high UCA1 expression group and low UCA1 expression group, number of patients with lymph node metastasis and distant metastasis, detection method of UCA1 expression levels, follow-up period and cut-off values, HRs, and corresponding 95% CIs for OS.

### Statistical methods

Data of pooled hazard ratios (HRs) were collected from the eligible studies; the log HR and standard error (SE) were used for aggregation of the survival results [17]. So as to evaluate the heterogeneity of the eligible studies, pooled HRs were executed using I^2^ statistics in this meta-analysis [18]. If the between-study heterogeneity was absent in the included studies (*p* > 0.1), we used fixed-effects model to analyze the results, while the random-effects model was applied for this meta-analysis when between-study heterogeneity was statistical (*p* < 0.1). The potential publication bias was assessed using the Egger's test. All the statistical analyses were carried out by using the Review Manager 5.3 and Stata 12.0. *p* values < 0.05 were considered statistically significant.
